# Elucidating the migrations of European seabass from the southern north sea using mark-recapture data, acoustic telemetry and data storage tags

**DOI:** 10.1038/s41598-024-63347-7

**Published:** 2024-06-08

**Authors:** Jolien Goossens, Mathieu Woillez, Serena Wright, Jena E. Edwards, Georges De Putter, Els Torreele, Pieterjan Verhelst, Emma Sheehan, Tom Moens, Jan Reubens

**Affiliations:** 1 Department of Biology, Marine Biology Research Group, Krijgslaan 281 - S8, 9000 Ghent, Belgium; 2 DECOD Ecosystem Dynamics and Sustainability, IFREMER, 1625 Route de Sainte-Anne, 29280 Plouzané, France; 3 Centre for Environment, Fisheries and Aquaculture Science, Pakefield Road, Lowestoft, NR33 0HT UK; 4 Coastal Systems, Royal Netherlands Institute for Sea Research, 1797 SZ Den Hoorn, The Netherlands; 5 Aquaculture and Fisheries Group, Wageningen University & Research, 6700 AH Wageningen, The Netherlands; 6 Sights of Nature, Vlamingenveld 89, 8490 Jabbeke, Belgium; 7 Flanders Research Institute for Agriculture, Fisheries and Food, ILVO Marine Research, Jacobsenstraat 1, 8400 Ostend, Belgium; 8 Research Institute for Nature and Forest (INBO), Havenlaan 88, Bus 73, 1000 Brussels, Belgium; 9 School of Biological and Marine Sciences, University of Plymouth, Drake Circus, Plymouth, PL4 8AA UK; 10 Flanders Marine Institute, Jacobsenstraat 1, 8400 Ostend, Belgium

**Keywords:** Animal migration, Behavioural ecology

## Abstract

The movement ecology of European seabass, *Dicentrarchus labrax*, remains poorly understood, especially in the northern ranges of its distribution. To investigate migration patterns of seabass from the southern North Sea, we combined data from different projects from four countries using various tagging techniques. This resulted in 146 recaptures (out of 5598 externally marked seabass), 138 detected animals (out of 162 seabass fitted with an acoustic transmitter) and 76 archived depth and temperature series (out of 323 seabass with an archival tag). Using geolocation modelling, we distinguished different migration strategies, whereby individual fish migrated to the eastern English Channel (15.1%), the western English Channel (28.3%), the Celtic Sea and the norther part of the Bay of Biscay (17.0%), or stayed in the North Sea (39.6%). A high number of seabass exhibited fidelity to the North Sea (90.5% of recaptures, 55.3% for acoustic transmitters and 44.7% of archival tags). Although seabass are generally considered to migrate southwards in winter, a large number of individuals (n = 62) were observed in the southern North Sea, where spawning might potentially occur in a particular deep location along the coast of Norfolk in the UK. Our results highlight the need to consider fine-scaled population structuring in fisheries assessment, and indicate that current seasonal fisheries closures are not aligned with the ecology of seabass in the North Sea.

## Introduction

Migration is a crucial aspect of fish ecology and entails the directional movement of individuals and populations from one location or habitat to another^1^. Migration enables fish to undertake different life-history stages in distinct essential habitats, e.g. for feeding or spawning^1,2^. The spatiotemporal change in habitat use shapes population structuring, as it determines the connectivity between conspecifics. Individuals can exhibit fidelity to the locations where they feed or spawn. In the case of spawning sites, fish can return to their natal breeding area (natal homing)^3^, or recurrently migrate to a specific site (other than their natal breeding area), potentially through learned behaviour^4^. A population can also display partial migration, whereby individuals exhibit different migratory patterns, with contingents being migrant and others resident^5,6^.

The highly mobile European seabass, *Dicentrarchus labrax* L., is distributed across the northeast Atlantic and the Mediterranean, which constitute separate genetic lineages^7^. In the northeast Atlantic, the seabass life cycle takes place in different habitats. After eggs are released in offshore spawning grounds, juveniles move to shallow areas in coastal lagoons, estuaries and rivers that serve as nurseries. Adult seabass (from the age 4–5 years and 32–36 cm length for males, and 5–8 years and 40–45 cm for females) feed in these inshore areas, as well as around offshore sand banks and ship wrecks, in the period starting from March–June to September–November. As temperatures drop, seabass aggregate in deeper, offshore waters for spawning from December to June, with the exact timing depending on the location and latitude^8–10^. The three main spawning areas are considered to be the Bay of Biscay (Rochebonne Plateau) and the western and eastern English Channel^2^. In contrast to the low genetic differentiation within the northeast Atlantic population, seabass movement patterns expose a complex population structure^11^. Individual seabass can reside in limited areas for long periods of time, and some exhibit interannual fidelity to both spawning and feeding areas^6,12–15^.

An area with particularly limited knowledge on seabass movements and habitat use is the North Sea. Seabass are known to occur in the coastal, estuarine and inshore areas along the Thames Estuary, Scheldt Estuary, Eastern Scheldt and Wadden Sea, which likely serve as nursery and feeding grounds^16–19^. Seabass marked near the Thames Estuary in summer were recaptured in the English Channel during spawning season^16^. Although seabass are generally considered to head southward for spawning, they may also spawn within the North Sea. In April and May 2011, stage 1 eggs (first 24 h) were found in the North Sea along the English coast, the Dogger Bank and the Voordelta (area stretching 3 to 15 km seaward along the Dutch coast from Walcheren to the Maasvlakte)^19^. Considering the temperature requirements for gonad development (minimum 9 °C for females), North Sea spawning is hypothesized to be possible during warmer years in the later months of the spawning season (April–May)^9,20^.

An essential tool to study (fish) movement ecology is tagging. The simplest and oldest tool consists of mark-recapture: a fish is captured and fitted with an external mark, after which a researcher depends on the recapture(s) of the animal to gain information on its movements. Fitting a fish with an electronic tag (internally or externally) vastly increases the information potential of an individual animal’s movement. In acoustic telemetry, an animal-borne transmitter emits an acoustic signal that can be detected when it is within the detection range of an acoustic receiver^21^. Detection data are accessed through the receiver and contain the timestamped information of the unique tag ID, potentially supplemented with a sensor measurement^22^. On the other hand, data storage tags (DST) store sensor information (e.g. depth and temperature) in the tag memory, requiring the physical recovery of the tag to access the stored data^23^. The resulting data series provide high resolution, continuous information on the depth and temperature experienced by the tagged fish, and can be used for geolocation modelling to reconstruct trajectories at a lower resolution (as a result of model error)^24,25^. To benefit from the highly complementary information of acoustic telemetry and DST, the two technologies can be combined in one physical tag^26^ or by double-tagging^27,28^. In this study, we combine mark-recapture, acoustic telemetry and DST data from seabass tagged in French, English, Dutch and Belgian waters to describe migration patterns of European seabass in the southern North Sea and adjacent water bodies.

## Results

### Mark-recapture

**Figure 1:**
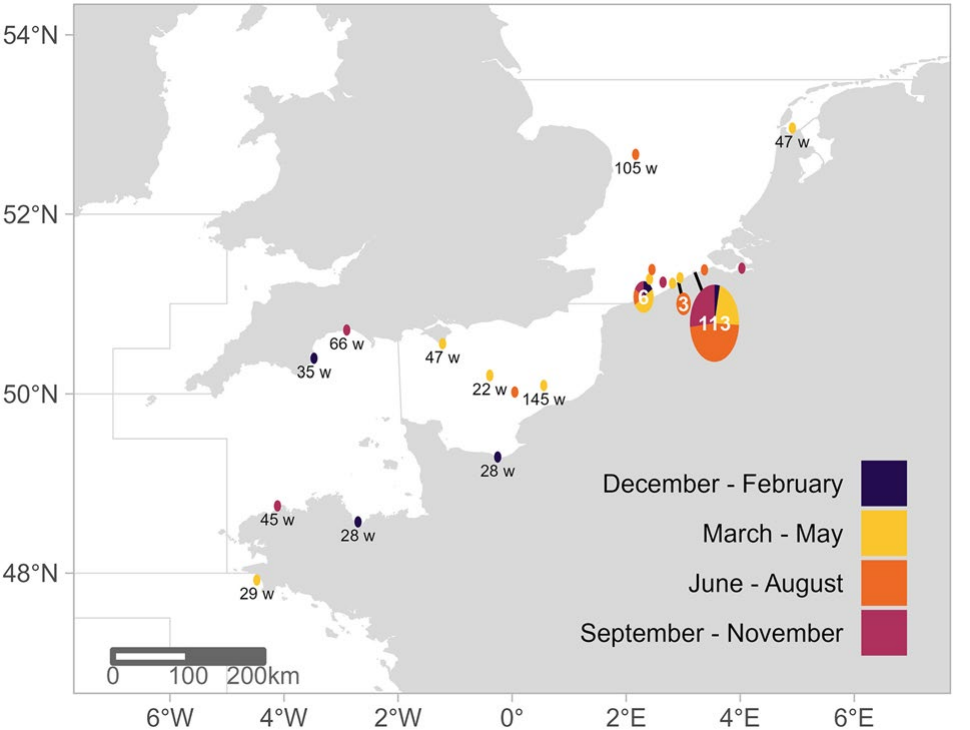
Recapture locations of marked seabass recaptures (white text), coloured by period of recapture. For long-distance recaptures (for which the exact recapture date was available), the time at large was displayed in number of weeks (w, black text). Grey lines depict the boundaries of ICES zones.

Out of 5598 marked seabass, 146 were recaptured (2.6%), of which 136 had both date and location information. The time period between capture and recapture (known for 137 seabass) ranged between one day and nearly four years (1392 days) with a median of 285 days. Positions of the 136 recaptures (Fig. 1) showed that 102 seabass (75.0%) were caught within a range of 5 km of the release location. Another 22 seabass (16.2%) were recaptured within 100 km distance of their respective release locations within the Belgian Part of the North Sea (BPNS), the Scheldt Estuary or Dunkirk. Twelve seabass (8.8%) were recaptured more than 100 km distance away within the southern North Sea, the English Channel and just south of the 48th parallel in the Bay of Biscay. Out of 84 seabass with at least 6 months at large, 76 were recaptured within the North Sea (90.5%).

### Acoustic detections

**Figure 2:**
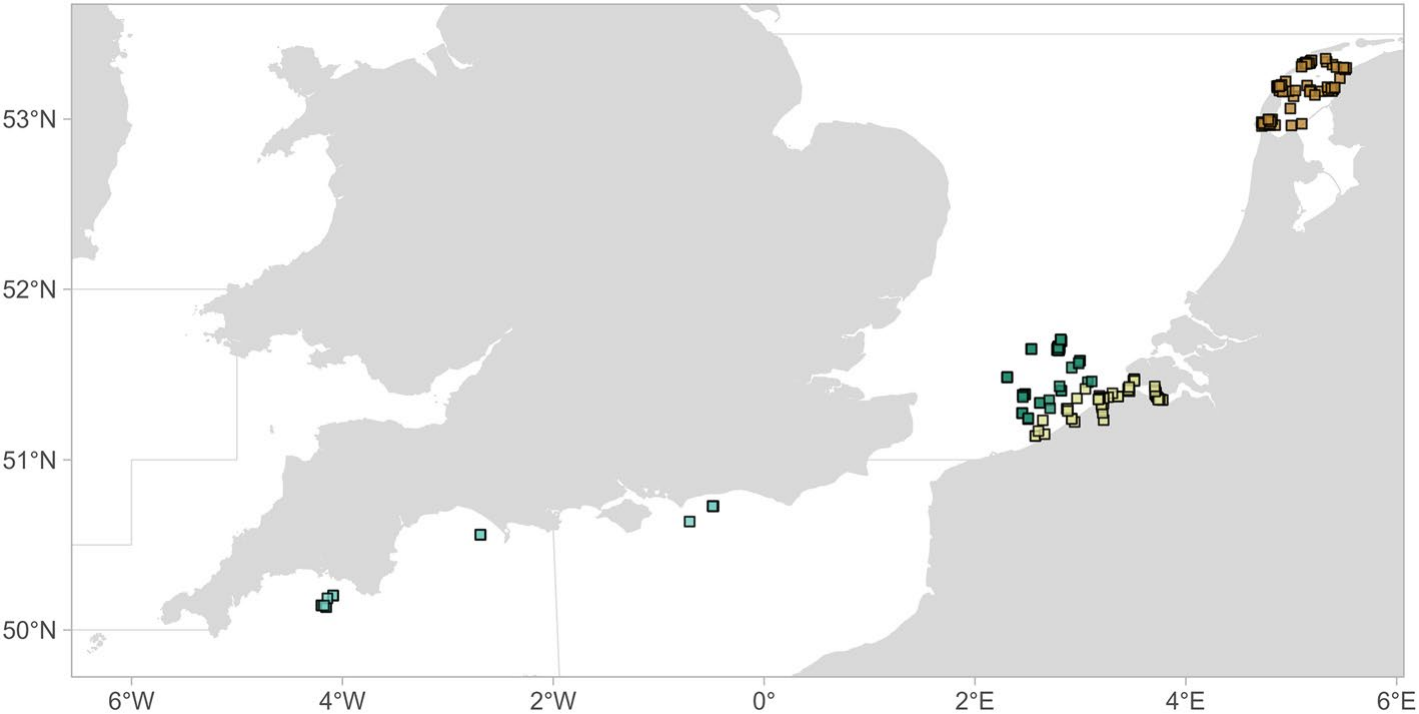
Locations of acoustic receivers with seabass detections, coloured by location (brown: Wadden Sea, dark green: offshore Belgian part of the North Sea (BPNS), light yellow: coastal BPNS and Scheldt Estuary, light blue: English Channel).

**Figure 3:**
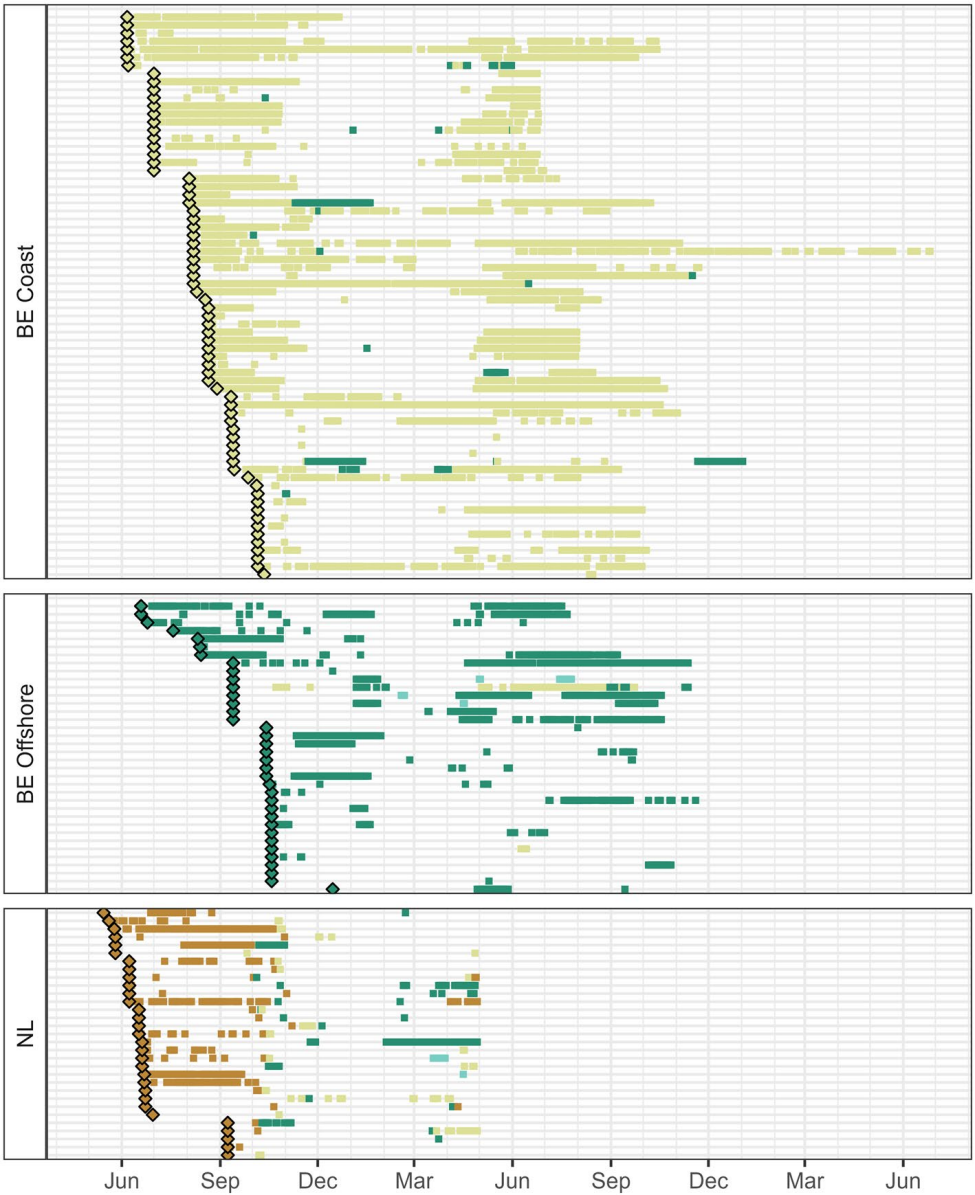
Abacus plot of acoustic data depicting a time line for individual tagged fish with the release (diamond) and detections (square) coloured by detection location (brown: Wadden Sea, yellow: coastal PBARN, green: offshore PBARN, light blue: English Channel). Fish were tagged from 2018 to 2022 and for each fish the detections were temporally aligned between tagging surveys using March 1 of the tagging year as a time reference.

Out of 162 seabass tagged with acoustic transmitters, 137 were detected for a total of 864,730 detections on 246 receiver stations (Fig. 2). Two fish died shortly after tagging and one tag experienced a hardware issue preventing acoustic signal transmission. These fish were excluded from the analysis. Fidelity to the North Sea (as defined in “Methods”) was observed for at least 85 animals (62.0% of detected seabass), whereas only three fish tagged in the Wadden Sea effectively returned to the same area (9.7%) (Table 1). The highest residency index (RI) was observed for fish tagged in Belgian coastal waters (median RI of 0.18 and 0.21 to the tagging area and North Sea, respectively). Lower values for RI were found for fish tagged offshore and in the Wadden Sea (median 0.01 to 0.04), but some fish were detected in the North Sea for approximately half of their time at large. Since large areas of the North Sea fell outside of the detection range of acoustic receiver stations, these values of RI should be regarded as underestimations. Fish tagged in the Wadden Sea were detected in offshore and coastal stations of the BPNS, but fish tagged in the BPNS or Scheldt Estuary were never detected in the Wadden Sea (Fig. 3). Fish tagged along the Belgian coast were detected on offshore stations, but only two seabass tagged offshore were detected along the coast. Five individuals (n_BE_ = 3, n_NL_ = 2) were detected along the English coast in the English Channel between February and July, on a network that was active since 2021.

**Table 1:** Acoustic telemetry results of site fidelity (seabass exhibiting fidelity out of the total number of detected animals), number of detection positive days (DPD, median [range]) and residency index (RI, median [range]) at receiver stations within the tagging area (TA) or the North Sea (NS).

TA	Fidelity-TA	Fidelity-NS	DPD–TA	DPD–NS	RI–TA	RI–NS
BE coast	46/70 (65.7%)	46/70 (65.7%)	67 [1–366]	71 [1–366]	0.18 [0.00–0.92]	0.21 [0.00–0.92]
BE offshore	20/36 (55.6%)	21/36 (58.3%)	21.5 [1–199]	24.5 [1–199]	0.05 [0.01–0.20]	0.05 [0.00–0.47]
Wadden	3/31 (9.7%)	18/31 (58.1%)	4 [1–109]	14 [1–111]	0.01 [0.00–0.32]	0.04 [0.00–0.33]

The pressure sensor measurements had a median depth of 7.8 m, with a maximum of 62.0 m registered in the English Channel. Temperature sensors registered a median of 15.2 °C, with minimum and maximum values of 2.8 °C and 28.4 °C, both registered in the secluded port area of Zeebrugge (discussed in more detail in Goossens et al.^29^).

**Figure 4:**
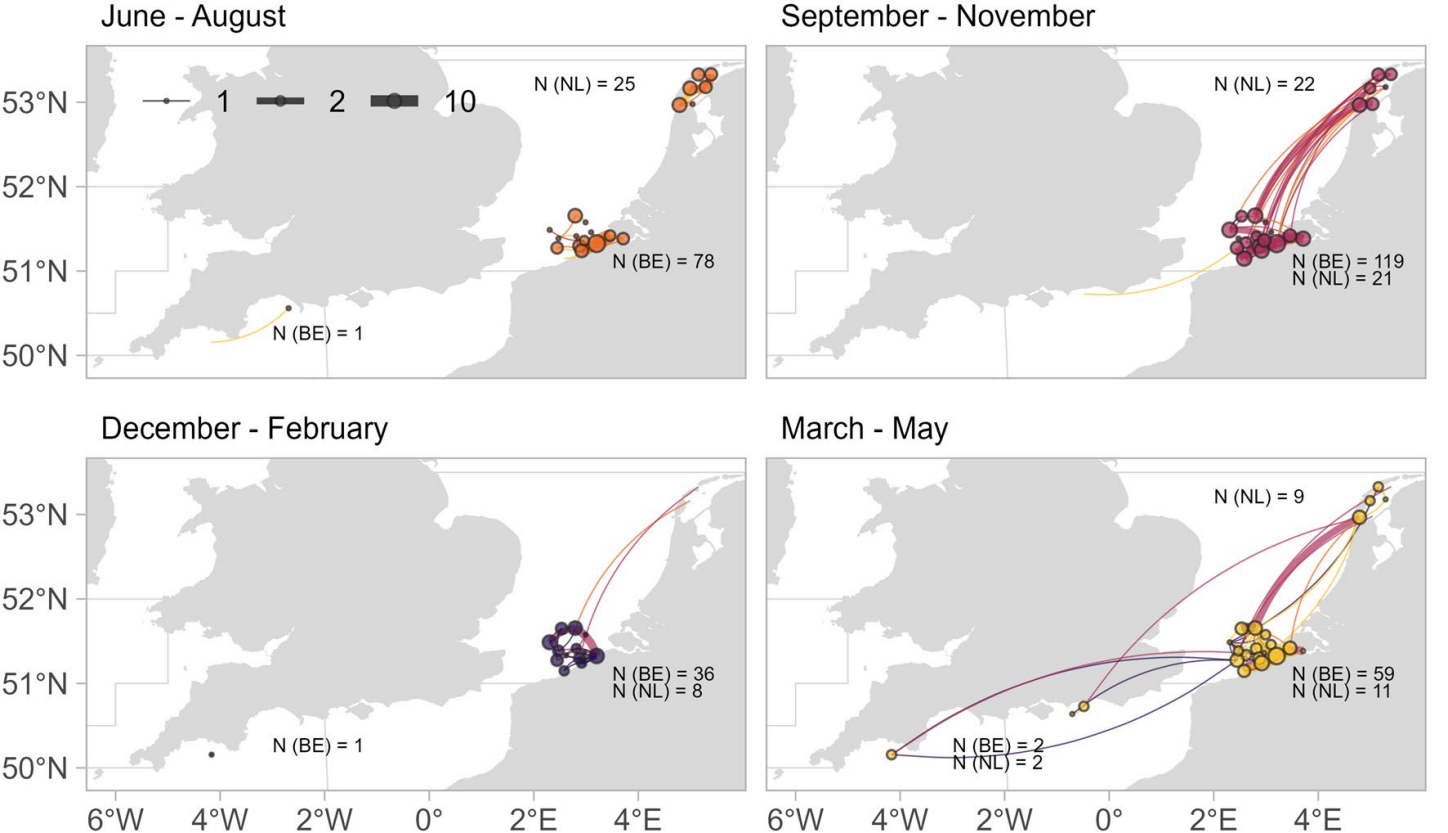
Spatial network maps for different periods: June–August (orange), September–November (pink), December–February (purple) and March–May (yellow). Nodes represent grouped receiver locations, are sized by the number of detected animals (n) and coloured by the period of detections. Edges represent frequencies of movement between receivers (right-hand curved from origin to destination receiver station) and are coloured by the period of detection at the origin station.

Throughout the study area, the largest number of seabass were detected from June to August (n = 92) and from September to November (n = 117) (Fig. 4), which were also the months when the majority of seabass were tagged. Seabass were observed to move between Dutch, Belgian and English receiver arrays mostly in the periods of March–May and September–November, with no large-scale movements registered within the periods of June–August and December—February. The lowest number of animals (n = 45) was detected from December to February, when most seabass were observed to have stayed at the port of Zeebrugge (n = 14), as well as around offshore wrecks and wind farms. From March to May, seabass (n = 80) were detected across the widest spatial range from the Wadden Sea to the western English Channel. The individuals detected in the western English Channel (n = 3) were never detected in the eastern English Channel, and vice versa (n = 2). Wadden Sea stations registered seabass throughout all seasons, except for December–February.

### Archival data

**Figure 5:**
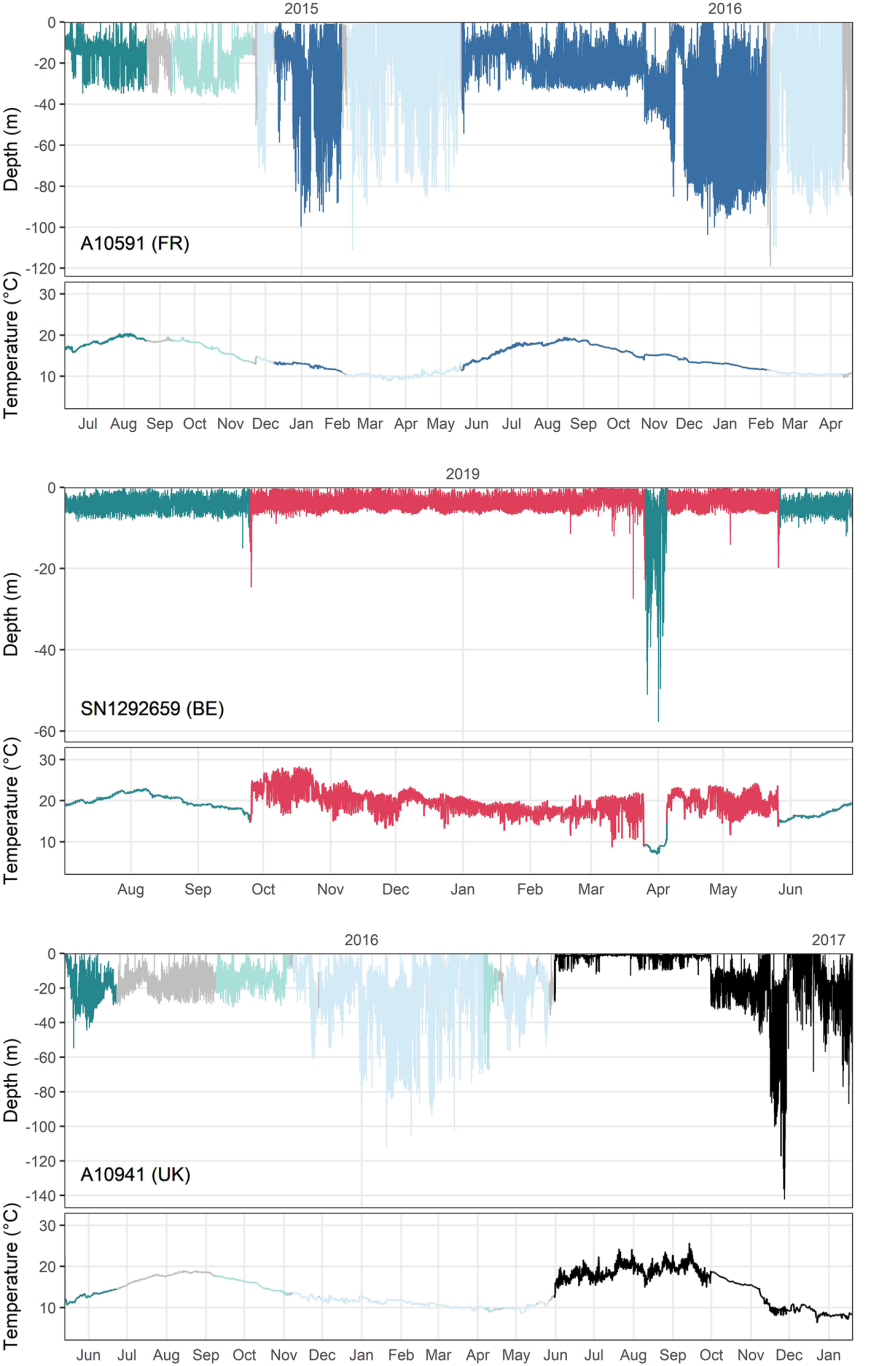
Exemplary time series of archived depth and temperature sensor measurements, coloured by the location of the daily position estimate (dark green: North Sea, light green: English Channel, light blue: Celtic Sea, dark blue: Bay of Biscay, red: cooling waters, grey: unsure, meaning daily position estimate of Viterbi track and mean or modal track were not in same area): an example of a seabass undertaking a migration (top), overwintering in cooling waters (middle) and showing shallow water occupancy with high temperature variability (bottom). For the latter, the black coloured data series were removed to estimate the trajectory.

**Figure 6:**
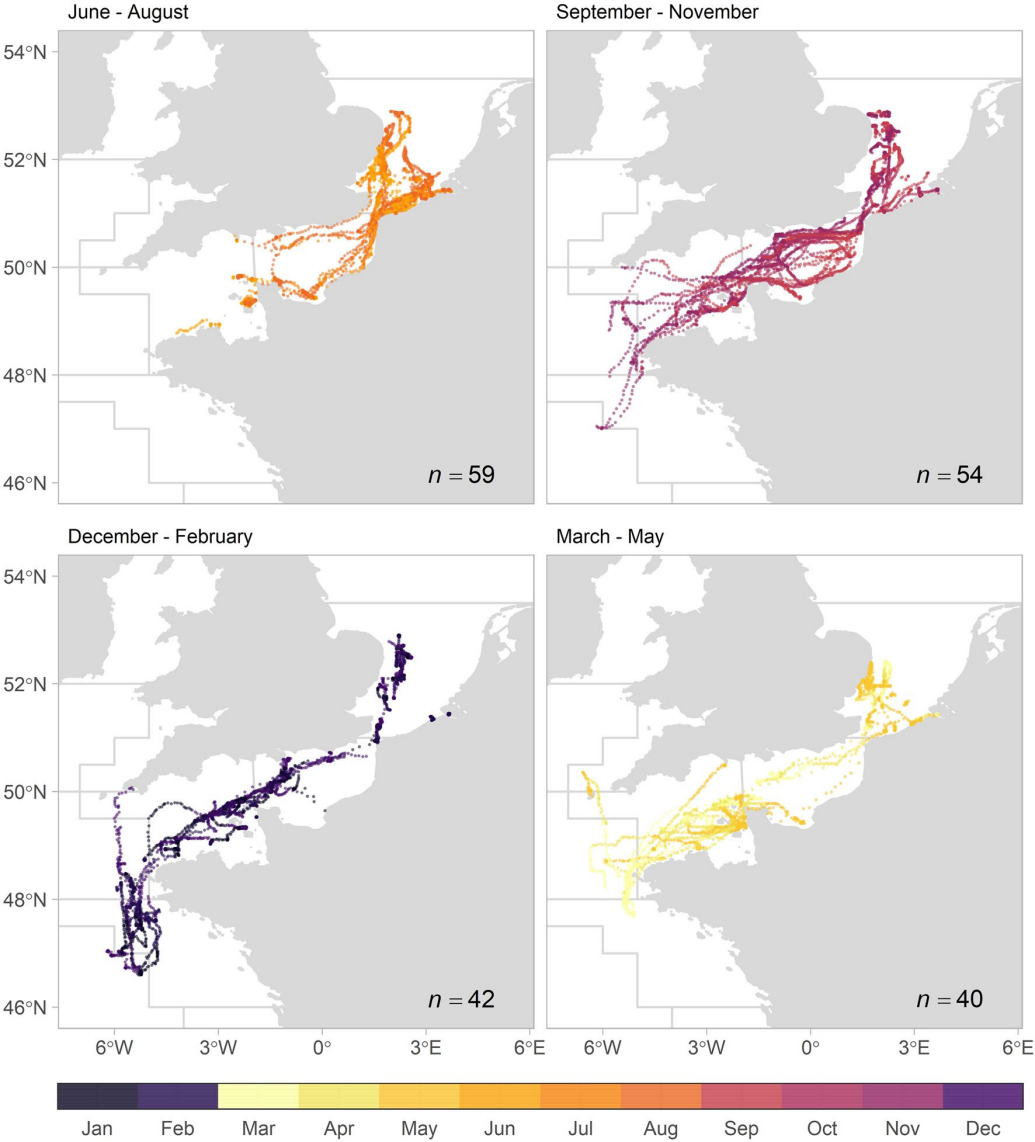
Daily position estimates derived from geolocation modelling of the archival data (Viterbi tracks, n = 62), coloured by month.

The geolocation modelling could be performed on 76 retrieved archival tags (n_FR_ = 48, n_UK_ = 18, n_BE_ = 10). Within the period that fish were evaluated to be alive, archived temperature measurements ranged between 4.3 and 32.3 °C, with a maximum depth of 173.5 m. The geolocation failed to converge for one tag and resulted in unreliable trajectories for six tags, producing 69 reliable trajectories with a median of 330 days (range 33–734 days). The higher resolution 3D-DCSM reference field could be applied to 31 data series, with the remaining 38 requiring the larger spatiotemporal range of the CMEMS-NWS model (see Methods section for details on geolocation reference fields). Behavioural switching could be applied to 17 tracks (24.6%). Eight out of ten recovered ADST rendered acoustic data, for which a detection likelihood was included. The warm temperature signal of power plant cooling waters was observed for 20 fish (28.6%), that spent a median 18 days in a warm water plume (range 4–235 days) (Fig. 5). Two fish experienced periods of high temperature variability in very shallow waters (Fig. 5), which we attributed to summer occupancy of inshore waters. Since the high variability of the temperature signal was not adequately represented in the temperature reference field, we only used the part of the data series before this behaviour for the geolocation. Out of the 69 estimated trajectories, 62 had sufficiently low error (distance between Viterbi track and mean or modal track: median 4.61 km, maximum 116.0 km) for spatial visualization (Fig. 6), but the remaining 7 were included in the temporal visualization (Fig. 8).

Seabass position estimates (n = 62) were located in the southern North Sea up to 52.9° N, the English Channel, the Celtic Sea and the Bay of Biscay up to 46.6° S (Fig. 6). From June to August (the period in which the majority of fish were tagged), seabass were mainly located in the North Sea and along the coast of the eastern English Channel. From September to November, seabass were widely distributed with high prevalence in the entire English Channel. From December to February, seabass were in the English Channel, as well as in the Celtic Sea and in offshore waters of the northern part of the Bay of Biscay. Seabass also resided in the North Sea during winter in inshore waters in a port area and cooling waters, as well as deeper locations off the English coast of Norfolk and Suffolk (around 52.5°N, 2.0° E). From March to May, seabass were mainly in the western English Channel around the Cotentin peninsula and Channel Islands, as well as in the North Sea.

**Figure 7:**
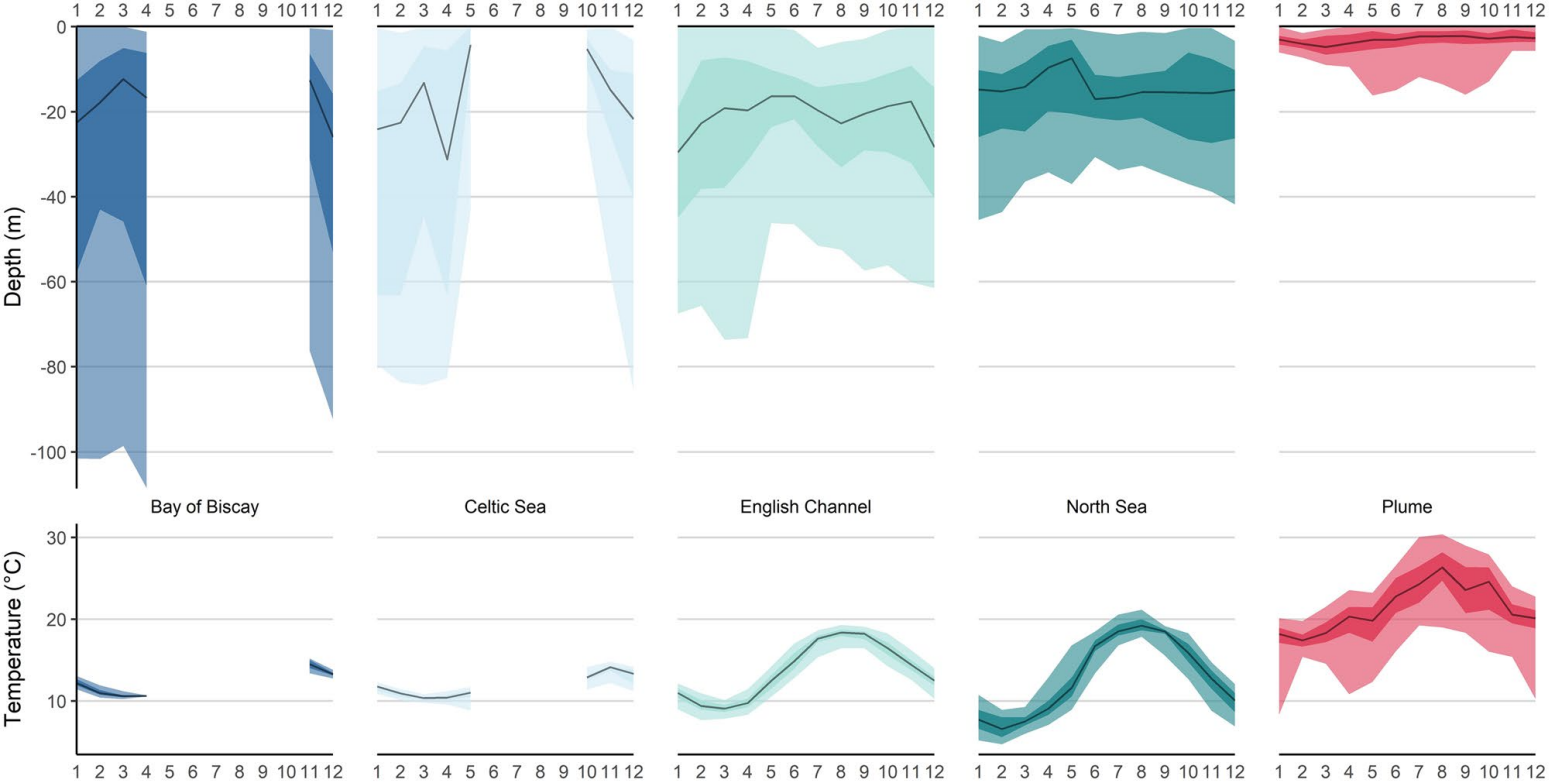
Plots of depth and temperature experience (black line: median; light and darker colouring: 95% and 50% confidence intervals, respectively) per month (x-axis), as experienced by seabass (n = 69) in different areas (dark green: North Sea, light green: English Channel, light blue: Celtic Sea, dark blue: Bay of Biscay, red: cooling waters).

Depth and temperature experiences varied in time and space (Fig. 7). In all areas, seabass went to greatest depths during winter. In the North Sea, median temperatures were below 9 °C from January to March. Temperature variance in the North Sea and English Channel was greater than in the Celtic Sea and the Bay of Biscay, but the greatest differences in temperature were recorded by seabass in cooling waters. Here, individual seabass would experience a median daily temperature change of 5.6 °C (with a maximum of 16.5 °C of temperature difference within one day) when entering/exiting the warm cooling waters.

**Figure 8:**
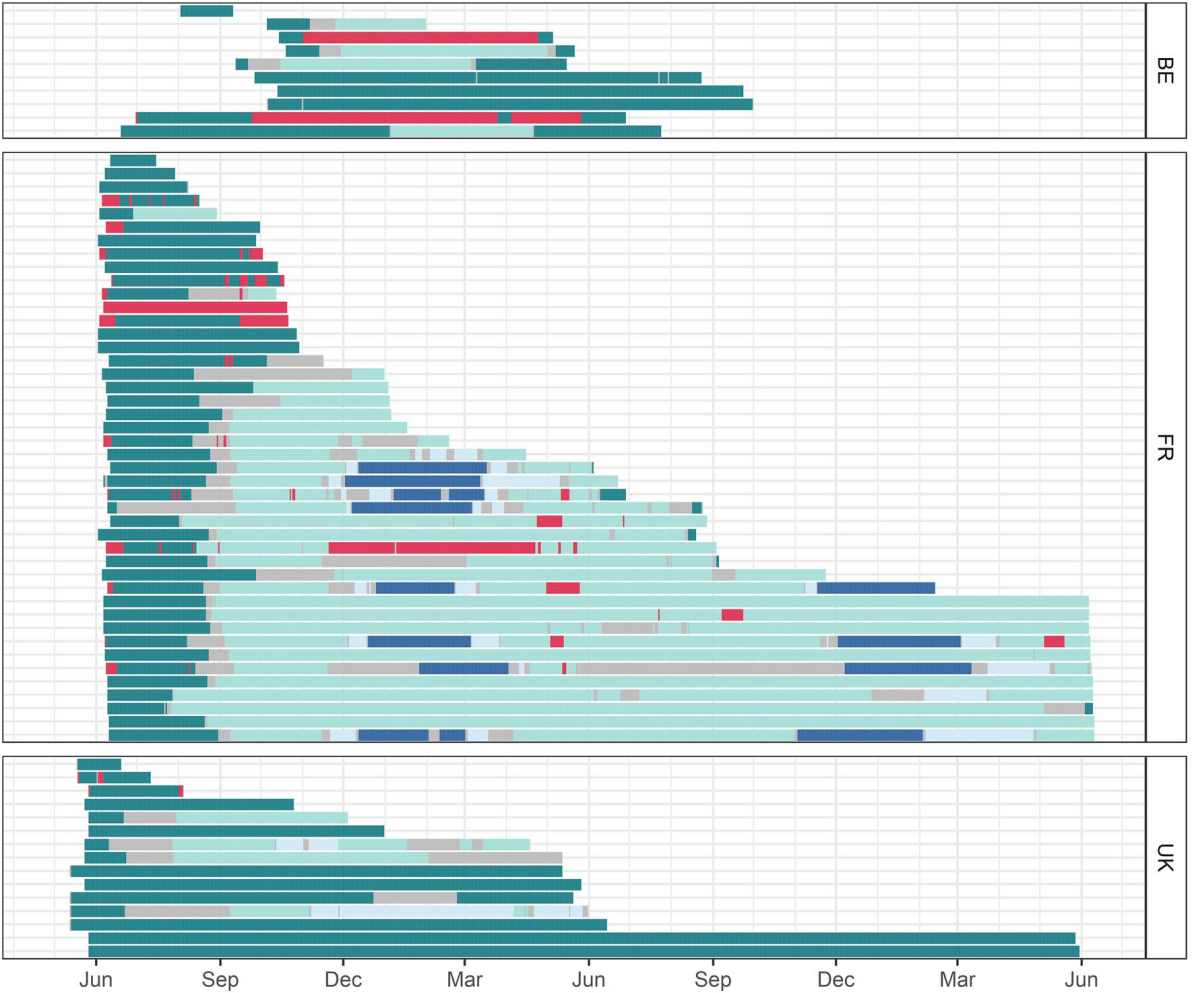
Abacus plot of archival data depicting a time line for the daily position estimates for individual tagged fish (n = 69), coloured by location (dark green: North Sea, light green: English Channel, light blue: Celtic Sea, dark blue: Bay of Biscay, red: cooling waters, grey: unsure, meaning daily position estimate of Viterbi track and mean or modal track were not in same area).

**Figure 9:**
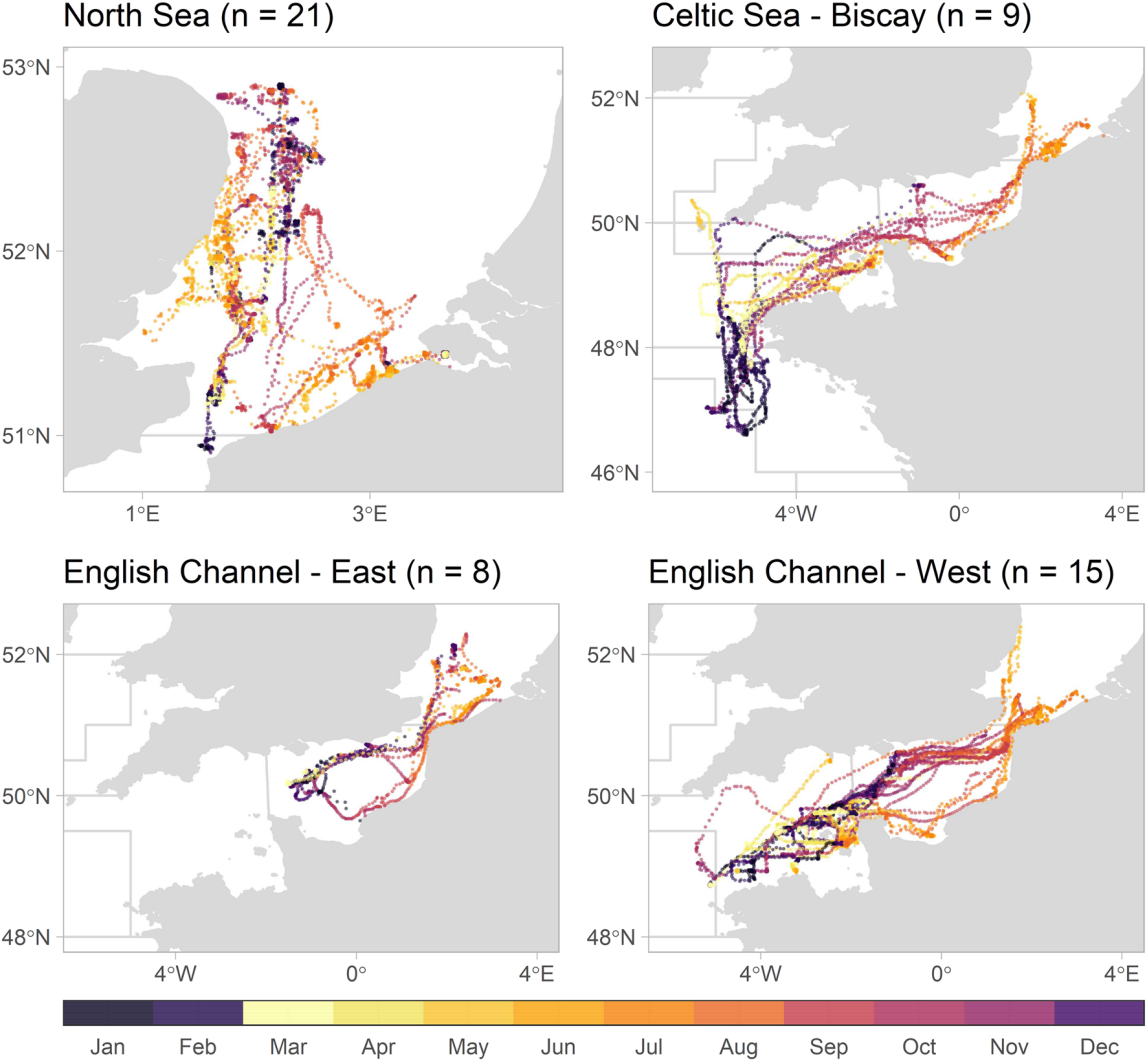
Maps of different migration strategies for seabass with data series over 90 days (n = 53), with daily position estimates coloured by month.

From the 47 fish with data series of at least 6 months, 21 (44.7%) exhibited fidelity to the North Sea (Fig. 8). Remarkably, all fish tagged along the coast of Dunkirk with data series longer than six months left the North Sea (between August and December, n = 28). Seabass tagged in the UK and Belgium either stayed in the North Sea or headed to the English Channel, with two fish going as far as the Celtic Sea. Migrations to the northern part of the Bay of Biscay were limited to seabass tagged in France (n = 8). Four different migration strategies or destinations were discerned for the seabass with data series over 90 days (n = 53) (Fig. 9): staying in the North Sea (n = 21), migration towards the eastern English Channel (n = 8), towards the western English Channel (n = 15) and towards the Celtic Sea and Bay of Biscay (n = 9). From the latter, four seabass returned to the North Sea, whereas the others went to the Bay of Biscay in winter, heading towards the southern coast of the English Channel in summer, and five seabass returning to the Bay of Biscay in winter. The largest estimated distance travelled was over 3000 km (Table 2).

**Table 2:** Overview of the migration strategies with the number of seabass (n) and the individuals’ distance travelled (km), estimated diffusion coefficient (D, km^2^/day), experienced depth (m) and temperature (°C) displayed as median [range].

Strategy	n	Distance (km)	D (km^2^/day)	Depth (m)	Temperature (°C)
North sea	21	580 [208–1732]	5.5 [2.0–30.0]	6.9 [0–70.1]	15.0 [4.3–28.3]
English channel E	8	840 [519–2663]	29.8 [16.1–90.0]	24.7 [0–90.5]	16.2 [6.9–20.8]
English channel W	15	1582 [1024–2000]	30.0 [6.0–62.4]	19.8 [0–173.5]	14.5 [6.5–29.3]
Celtic Sea—Bay of Biscay	9	2496 [1648–3089]	61.2 [30.0–91.0]	17.3 [0–123.6]	14.1 [8.1–29.7]

## Discussion

In this study we characterized the migration patterns of European seabass, tagged in the southern North Sea using mark-recapture, acoustic transmitters and/or archival tags. By combining data from projects applying distinct tagging technologies we could overcome some of the limitations of either technology. Specifically, the continuous archival time series allowed the reconstruction of large-scale migration trajectories, whereas mark-recapture and acoustic telemetry data provided ground-truth information on the presence of seabass.

### Migration strategies

European seabass tagged in the southern North Sea were found to inhabit an area extending from the southern North Sea to the northern Bay of Biscay (west of Brittany). In summer, seabass resided in the North Sea and along the coast of the English Channel. For the rest of the year, seabass were spread across the entire area, with high movement activity from September to November. Fidelity of seabass to the North Sea was seen for almost half of archival tags and more than half of acoustic tags.

Our results highlighted that individual seabass displayed distinctly different seasonal space use patterns, which we described as different migration strategies. The reconstructed trajectories from longer data series (over 90 days) indicated that many seabass stayed in the North Sea (n = 21) or migrated to the English Channel (n = 23), while some went as far as the Celtic Sea and the northern part of the Bay of Biscay (n = 9). Seabass from the Wadden Sea, tagged with acoustic transmitters, headed southwards to the Belgian EEZ and the English Channel, but the opposite movement was registered for only one individual (mark-recapture data).

The different strategies raised the question why a seabass would migrate over 3000 km when conspecifics travelled hundreds to thousands of kilometres less. Dambrine et al.^2^ showed that environmental covariates served as poor predictors for seabass spawning aggregations, suggesting other mechanisms may be at play, such as natal homing or learned behaviour. Natal homing would mean seabass return to the area where they hatched as an egg^14^, whereas, if driven by learned behaviours, they would be expected to follow other (older) adults^4^. Migrations to the northern Bay of Biscay and the Celtic Sea (n = 11) were performed by seabass tagged in 2014 and 2015 (in French and UK waters), whereas fish tagged from 2016 onwards (n = 11) stayed in the North Sea or migrated to the English Channel. This may be due to the small sample size or the particular environmental conditions of these years, but could also suggest that seabass travelled longer distances in the past, but have migrated less southward in more recent years. This could potentially be explained by increased temperatures in the past decade that might have made habitats closer to home more suitable as wintering or spawning areas^3^. Another explanation may be that the knowledge of southward migration routes was lost as a result of high fishing pressure that depleted local seabass schools, including the older individuals who ‘knew the way’^4,12^. If natal homing was at play, it could be possible that recruitment to the North Sea from spawning aggregations in waters west of Brittany has been poor in past years due to hydrodynamic conditions^20^, and that, in more recent years, adult seabass in the North Sea originated from spawning migration strategies closer to the North Sea.

Out of 36 seabass tagged offshore (more than 6 nautical miles from the coast), only few were sporadically observed along the coast: Three seabass were observed (two fish detected acoustically and one through its reconstructed trajectory) in coastal waters of the North Sea and another three were detected in coastal waters of the English Channel. Until now, seabass tagging research always involved individuals captured in coastal locations^12,13,15,30^. The generally assumed movement pattern of seabass—spawning in offshore locations during winter, but heading towards the coast in summer—may therefore have been biased by the logistical preference for coastal tagging locations. Ongoing tagging efforts in offshore locations (in the context of the FISH INTEL project) will elucidate if seabass from offshore areas exhibit significant connectivity to the coast or if they undertake distinctly different movements than coastal seabass.

### Potential spawning area in the North Sea

Several seabass from the tagging areas in Suffolk, Dunkirk and Belgian waters moved towards offshore areas and coastal waters of Suffolk and Norfolk in the UK, which was mostly the case for seabass that stayed in the North Sea throughout the year. In particular, trajectories passed through a deep location near the coast of Norfolk and Suffolk (around 52.5°N, 2.0° E) throughout the year, which was further supported by a seabass mark-recapture (marked with a Pederson disc) in this area during the summer of 2021 (Fig. 1). Although it is not known what a seabass spawning event looks like in terms of vertical movement behaviour^31^, the presence of 7 seabass in this area during the potential spawning period indicates this deep spot in the North Sea may serve as a spawning area. This is further supported by a study in 2011, where seabass eggs were observed in the North Sea^19^. The temperature sensor measurements indicated North Sea spawning would have only been feasible from April onwards, since temperatures below 9 °C would hamper female seabass gonad development^9^. To better understand seabass behaviour in the North Sea, the location off the English coast of Norfolk and Suffolk (around 52.5° N, 2.0° E) would be a key position for an acoustic receiver array.

### Complementary tagging techniques

The description of seabass migration patterns in this study greatly benefited from applying different tagging techniques. The continuous data series from the DST allowed for the geolocation of entire trajectories, with the drawback that the modelled positions remained estimates rather than observations. Experts must therefore remain critical of the results of the geolocation model, which are useful, but cannot be considered as true paths. Acoustic data allowed to investigate fish movement in coastal areas where temperature reference fields might fail (e.g. the two fish with high temperature variability in inshore waters, Fig. 5). Acoustic data could validate trajectories of fish, either directly for which both data types were available^26,27^, or by corroborating the possibility of certain migration headings and destinations. The latter was also true for conventional tags: Even though mark-recapture provides less data on a lower number of tagged fish (2.6% recaptures in this study and e.g. 4.5% reported by Pawson et al.^32^), these data were highly valuable for ground-truthing model outcomes. Moreover, the mark-recapture project involved the collaboration of volunteer anglers who promoted the research in their networks, which we believe to have contributed to the notification of recaptures of both conventional and electronic tags.

### Implications for fisheries management

The findings of this study are relevant for fisheries management and stock assessment. European seabass of the Northern stock experienced critical declines in the past fifteen years due to high fishing pressures and poor recruitment^33^. The current stock delineation originated out of management practicalities, because of lacking biological information to substantiate stock structure^34^.

Our data contested previous indications that the North Sea might consist of a separate unit^32^, although many seabass were seen to reside in the North Sea throughout the year. Eight seabass from the southern North Sea were shown to cross the boundaries of the Northern stock into the range of Bay of Biscay stock, although these migrations were not observed in recent years. The variability in individual migration strategies supported a high degree of fine-scaled population structuring, with previous research demonstrating the existence of separate entities (during the feeding season) distanced only a few kilometres from each other^29^. These migration strategies could be included in population dynamics and stock assessment models^35^. Aside from optimizing existing models, this fine-scaled population structure should be accounted for when assessing the uncertainty of the relationship between fishing pressure F and stock biomass SSB^36^. The effects of fisheries locally depleting groups of seabass and potentially erasing certain movement strategies on the stock unit or population as a whole remains poorly understood^4^.

The individual variability in spatiotemporal habitat use should also be accounted for in fisheries management. Ever since 2016, EU fisheries measures include a seasonal closure for commercial seabass fishing in February and March to protect spawning aggregations^37,38^. Considering the different migration strategies, only a fragment of the population would be effectively protected during their spawning time by these measures. For seabass residing throughout the year in the North Sea, the seasonal closure would have to be in April and May. Moreover, the complexity in the structuring of the seabass population shows that these animals are not sufficiently understood to apply the principles of ‘economic rationality’ that seek to maximize yield^39^.

## Methods

### Study area

**Figure 10:**
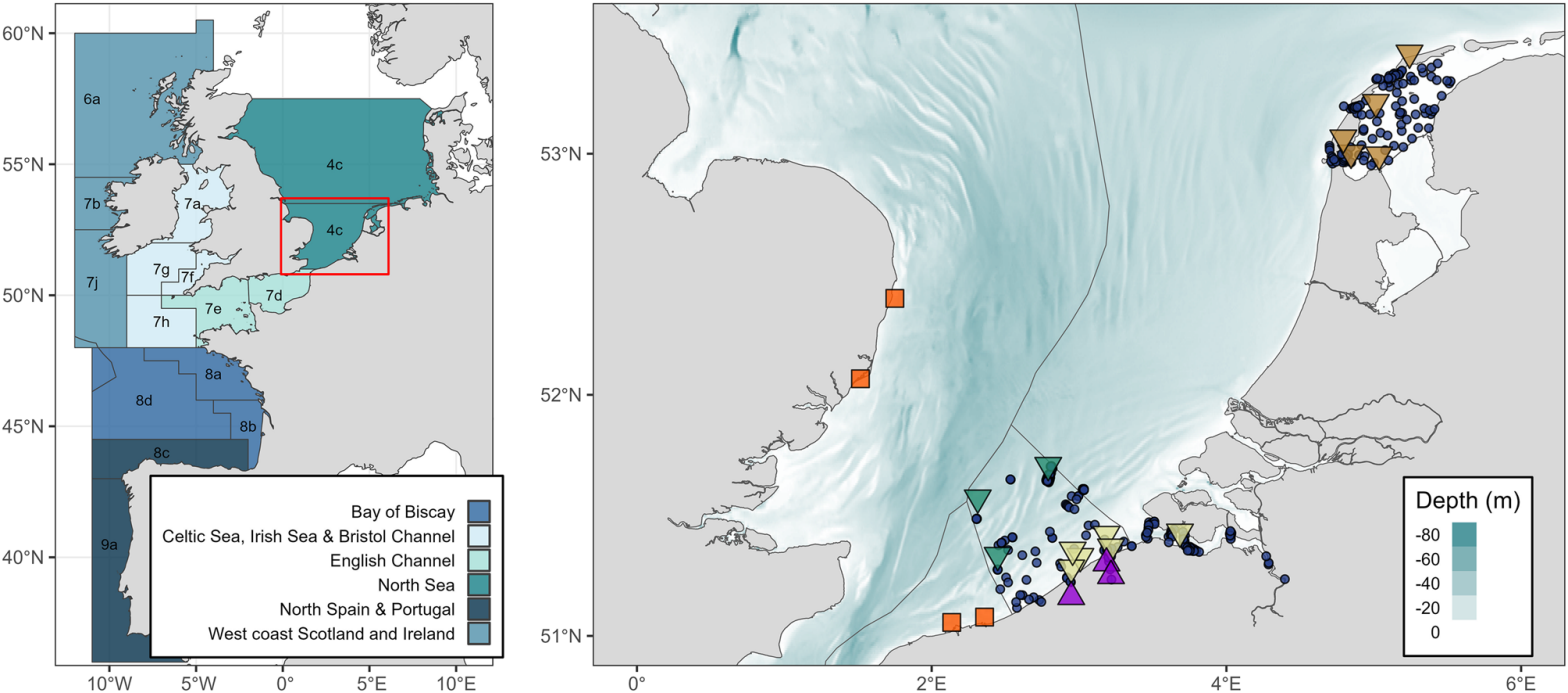
Left: Current ICES stock division in the northeast Atlantic: the Northern Stock (central and southern North Sea + English Channel + Celtic Sea, Irish Sea and Bristol Channel: 4b,c, 7a,d–h), the stock of west coast Scotland and Ireland (west of Scotland, west of Ireland and eastern part of southwest of Ireland: 6a, 7b,j), the Bay of Biscay stock (northern and central Bay of Biscay: 8a,b. Note that 8d was also marked in blue, but is strictly seen not included in stock) and the stock of North Spain and Portugal (southern Bay of Biscay and Atlantic Iberian waters: 8c, 9a). Right: Map of tagging locations (purple: external tags; orange: DST; brown: acoustic, Wadden Sea; light yellow: ADST/acoustic, Belgian coast & Scheldt Estuary; dark green: ADST/acoustic, offshore) and acoustic receiver locations (blue dots) in the southern North Sea. Bathymetry data originate from the General Bathymetric Chart of the Oceans (GEBCO, 2014).

The study area consisted of the southern North Sea (or Southern Bight; ICES division 4c) and its connected water bodies (Fig. 10). The complex hydrodynamics are influenced by strong tidal currents, saltwater inputs from the English Channel and freshwater inputs from rivers such as the Thames and Scheldt^40^. The overall shallow area (maximum depth 91 m) is mainly characterized by sand banks, with seabed substrate being highly impacted by bottom trawling^41^. Hard substrate habitats now mainly consist of man-made structures, such as ship wrecks and wind turbine foundations^42^. The southern North Sea falls within the exclusive economic zones of the United Kingdom (UK), The Netherlands, Belgium and France and is heavily influenced by anthropogenic impacts (e.g. overexploitation and climate change), whereby the North Sea fish community has undergone pronounced spatiotemporal changes in composition^43^. Regarding fisheries management and assessment, seabass in the North Sea are classified as the Northern stock (central and southern North Sea, Irish Sea, English Channel, Bristol Cannel and Celtic Sea; ICES divisions 4b,c, 7a,d–h)^44^ (Fig. 10). The other stocks in the northeast Atlantic consist of north Spain and Portugal (southern Bay of Biscay and Atlantic Iberian waters; ICES divisions 8c,9b), the Bay of Biscay (northern and central Bay of Biscay; 8ab) and west coast Scotland and Ireland (west of Scotland, west of Ireland and eastern part of southwest of Ireland; 6a,7b,j)^34^.

### Tagging

Tagging data originated from different projects over different time spans, using different methods of conventional and electronic tagging (Table 3, details on tag settings in Additional information S1). Fish were caught from boats or from shore at different locations along the Belgian, British, Dutch and French coast in the southern North Sea (Fig. 10). Seabass were captured with hook and line, gillnets and fykes, and one seabass was caught with a longline. For angling, mostly artificial bait was used (wobblers and soft lures), except for some offshore and estuarine locations where live bait was used (ragworms, lugworms and crabs). Between 2006 and 2021, 5598 seabass were fitted with Pederson discs (9.5 mm diameter; Floy Tag & Mfg. Inc., USA) along the Belgian coastline with the majority of captures coming from the Port of Zeebrugge. All electronic tags were surgically inserted in the abdominal cavity. Data storage tags (DST) of the type Cefas G5 2 Mb DST with a floatation collar (Cefas Technology Limited, UK) were used on 150 seabass caught off the French coast of Dunkirk in June 2014, and on 64 seabass caught in English waters along the coast of Suffolk in May 2015 and 2017. Acoustic tags of the types V9P, V13, V13AP and V16 (69 kHz, MAP114, protocols A69-9001, A69-9006, A69-9007, A69-1602; Innovasea Ltd., USA) were used on 22 seabass in Belgian waters between June 2018 and September 2020 and on 214 seabass in Dutch waters between May 2021 and September 2022. Acceleration sensor measurements of the V13AP transmitters were not used, as this information was outside of the scope of this study. Acoustic data storage tags (ADST; ADST-V9TP: 69 kHz, MAP114, protocol A69-9006; Innovasea Ltd., USA) were used on 109 bass in Belgian waters between 2018 and 2021. Seabass were tagged in coastal locations (less than 6 nautical miles from the shoreline) in Dutch, English, French and Belgian waters, as well as in offshore locations in Belgium (acoustic and ADST). Tagging was performed in accordance with the guidelines for animal experiments for the relevant national authorities under the ethical certificate and license numbers 01987.02 (France), EC2017-080 (Belgium), PPL 70/7734 (UK), AVD401002016613 and AVD40100202114609 (The Netherlands). Tagging procedures were explained in full detail in other publications that used these data sets^15,26,45,46^.

**Table 3:** Number of seabass (N) and their length (median [range]), tagged with different tag types in different tagging areas.

Tag type	Tagging area	Tagging year	N	Length (cm)
Pederson discs	BE coast	2006–2021	5598	31.5 [9.0–81.0]
DST	FR Dunkirk	2014	150	51.7 [43.2–69.1]
	UK Suffolk	2014–2016	64	58.5 [49.0–76.0]
Acoustic	Wadden Sea	2021	31	51.6 [40.0–75.0]
Acoustic + ADST	PBARN coast	2018–2021	79	46.0 [34.0–74.0]
	PBARN offshore	2018–2021	52	47.0 [33.0–57.0]

Data collection differed between technologies. For the acoustic tags (including ADST), the tag ID and sensor information were transmitted (69 kHz, MAP114, protocol A69-9006) to acoustic receivers (VR2W, VR2AR and VR2Tx; Innovasea Ltd., USA) of permanent and temporary networks in the study area^45,47^. From previous range testing in the BPNS, the median detection range distance (with 50% probability of observing the presence of a tagged seabass within a day’s time) was estimated at 566 m^48^. Acoustic data and metadata was managed through the online database of the European Tracking Network (ETN; https://lifewatch.be/etn/), enabling direct access to detection data on other receiver arrays included in ETN. At the time of writing, detection data from fish tagged in the Wadden Sea were still being collected and were under limited disclosure. This dataset was therefore limited to a subset of the detections up to 30 April 2022 of 31 individuals which were detected by arrays outside of the Wadden Sea. For conventional tags, we relied on voluntary reporting by people encountering the marked fish (mainly fishers), who were asked to report the ID, date and location of recapture (and if possible: the length and weight of the recaptured seabass). To access archival sensor information, DST and ADST had to be recovered. To increase the recovery, floatable tags were used that could drift ashore if separated from the fish. For both conventional and archival tags, tag return was incentivized with rewards, ranging from 2 to 100 euro depending on the project and tag type. The tagging experiments of the different projects were publicised through various media, including posters, flyers, emails to fisheries and stakeholders, and articles in (mainly angler specific) websites and magazines.

### Data processing

For the mark-recapture data, we calculated the distance between the release and recapture position, as well as the number of days between the two events. Some observations could not be used for these calculations, as they were communicated vaguely in terms of time (e.g. the month or year of recapture) and location (e.g. the EEZ). Telemetry data were analysed after a quality check. If an animal was detected only once on a receiver array, that detection was considered unreliable and hence removed. For long-distance movements (more than 100 km distance between subsequent detections), we evaluated whether the movement was feasible (e.g. a single detection implying a movement of more than 100 km distance both back and forth within the same day was removed). A residence index (RI) was calculated to quantify daily presence in the North Sea and in the tagging area (Wadden Sea, coastal BPNS or offshore BPNS). If a seabass was detected at least once in a day, that day was considered as a detection positive day (DPD). The RI was then calculated as the number of DPD out of the time at large, the period from the tagging event to the end of the battery lifetime or recapture of the fish (if that date preceded the end of battery lifetime). For some of the archival depth series, we could see that the depth sensor experienced drift, whereby depths strayed from a minimum of 0 m. To correct for depth drift, time series were processed by using a running minimum over a 7-day period.

For all tagging techniques, a fish was considered to exhibit fidelity to the North Sea if it was observed (through recapture, acoustic detection or trajectory reconstruction, see below) there for at least 180 days (6 months) after the tagging event. For acoustic tags, we also calculated site fidelity to the area of tagging (Wadden Sea, coastal BPNS or offshore BPNS).

### Geolocation

From archival data, trajectories were reconstructed with geolocation modelling using a hidden Markov model (HMM)^49^. As the model was fully described in previous publications^15,26^, we limit the explanation here to the alterations made. The choice of temperature reference field was based on the necessary spatial extent of the estimated trajectory. The 3D Dutch continental shelf model in flexible mesh (3D DCSM-FM) had a high spatial resolution (North Sea and coastal waters: 0.5ʹ × 0.75ʹ, English Channel: 1ʹ × 1.5ʹ; latitude × longitude)^50^, but a limited spatial range (48.8° N–53.0° N, 3.2° W–5.0° E). The Atlantic Ocean Physics Reanalysis for the European North West Shelf (CMEMS-NWS Physics) model had a greater spatial range (which we limited to 44.0° N–56.0° N, 7.0° W–7.0° E), but a lower resolution (0.067° × 0.111°, latitude x longitude). 3D DCSM-FM temperature data were available for the years 2014–2016 and 2018–2020, whereas CMEMS-NWS Physics data were available for all years (since 1993) up to 30 June 2022 at the time of writing. If the spatiotemporal range of the trajectory allowed for it, we opted for the higher resolution model 3D DCSM-FM and otherwise we used CMEMS-NWS. The 3D DCSM-FM included bathymetry data, but we used the General Bathymetric Chart of the Oceans (GEBCO, 2014; resolution 0.5ʹ × 0.5ʹ). For the ADST resulting in both acoustic detections and archived sensor information, the detection likelihood was included^26^. Using the archival depth series, daily activity states were identified as low or high activity using a HMM^31^. The activity state identification was evaluated by visually checking in the archival depth series whether the low activity state did not include high activity vertical movements. If the states were considered reliable and if the model converged, the behavioural switch was included in the model^15,24^.

We calculated trajectories as the most probable sequence of positions from the daily posterior probability distributions, using the Viterbi algorithm^49^. Goossens et al.^26^ showed the estimation of seabass tracks performed with a median accuracy of 21.4 km (maximum 134.7 km) of daily position estimates. As an additional validation, we calculated the distance between the daily position estimates of the Viterbi track with those of the maximum posterior mode and mean posterior tracks, as detailed by Woillez et al.^49^. A track was considered reliable if the median error was below 50 km and the maximum below 120 km (respectively the average errors for demersal and pelagic fish geolocation models^25^). Tracks with median errors over 50 km or maximum error between 120 and 240 km were not visualized spatially, but they were included in temporal representation (see below).

### Data visualisation

Data from all tagging techniques were visualized on a spatial and temporal dimension. To distinguish a relevant seasonal component, we explored data from all techniques in monthly time frames to determine which months were similar in space occupancy. Based on these explorations, we grouped observations into the seasonal component December–February (winter), March–May (spring), June–August (summer) and September–November (autumn). Recaptures with both date and location information were visualized as recaptures per season on a map. Acoustic detections of every tag ID were visualized over time in an abacus plot. Spatial visualizations of the acoustic telemetry data included a map with the locations of detections, as well as seasonal spatial network maps. For the latter, stations were grouped to calculate the number of detected animals and the counts of directed movements between different areas^51^.

The daily position estimates derived from geolocation modelling of the archival data, were overlaid on ICES divisions to define whether a seabass was in the area of the North Sea (4b,c), the English Channel (7d,e), the Celtic Sea (7a,f,g,h) or the Bay of Biscay (8a,b,d). If the daily position estimate from the Viterbi track was located in another area than the estimate of the maximum posterior mode or the mean posterior track (see above), we considered the area of location of a seabass for that day as unknown. We visualized examples of archival depth and temperature series, as well as the depth and temperature experience (median, 50% and 95% confidence intervals) per area. An abacus plot visualised the daily area estimates over time for every tag ID. Daily position estimates of tracks (with median error was below 50 km and the maximum below 120 km, see above) were spatially visualised per season and per identified migration strategy.

Aside from the geolocation model, which was run in Python 2.7^52^, all analyses and visualizations were performed in R software^53^.

### Supplementary Information

Supplementary Table S1.

## Data Availability

Data available upon request to the corresponding author.
