# Supplementary material for: Elucidating the migrations of European seabass from the southern north sea using mark-recapture data, acoustic telemetry and data storage tags
Source: Sci Rep. 2024 Jun 8;14:13180. doi: 10.1038/s41598-024-63347-7 (PMC11161488; doi:10.1038/s41598-024-63347-7)
Supplement: Supplementary file 1 — Supplementary Table S1. [file 41598_2024_63347_MOESM1_ESM.pdf]

SUPPLEMENTARY INFORMATION

Table S1 - Tag settings applied for external (Floy Tag & Mfg. Inc., USA), archival (CTL, UK), acoustic and acoustic data storage tag types (Innovasea Ltd., USA) for a number of tagged animals (N). Acoustic and archival tags transmitted and / or logged temperature (T), acceleration (A) and pressure (P) sensor measurements. The number of tags of each type was shown for ever. For estimated battery life, it should be noted that the ADST-V9TP was programmed to effectively stop transmitting after the set number of days, whereas the V13AP and V9P could continue transmitting as long as there was battery left. Signals were transmitted at predefined settings within a step of a fixed number of days at high (H) or low (L) transmitting power output between a minimum and maximum interval. Power output was 158 dB for V16, 152 dB for V13, 154 dB for V13AP, 151 dB for ADST-V9TP and 146 (L) or 151 (H) dB for V9P.

Tag type	Model	N	Country	Tagging period	Battery life (days)	Logging interval T–P(s)	Transmitting settings
External	Pederson disc	5598	BE	2006 – 2021	-	-	-
Archival	Cefas G5	150	FR	2014	730	90 - 90 (680 days);<br>180 - 180 (50 days)	-
	Cefas G5		UK	2014 – 2016	730	-	-
Acoustic	V13AP	11	BE	2018 - 2020	495	-	H 495 days (140 - 220 s)
		1	BE		198	-	H 30 days (30 - 60 s);<br>H 168 days (140 - 220 s)
		2	NL	2021	278	-	H 278 days (50 – 100 s)
		3	NL	2021	293	-	H 293 days (50 – 100 s)
	V13	19	NL	2021	915	-	H 235 days (135 – 235 s)
	V16	8	NL	2021	1415	-	H 80 days (40 – 80 s)
	V9P	10	BE	2020	502	-	H 153 days (180 - 280 s);<br>L 120 days (300 - 400 s);<br>H 229 days (300 - 400 s)
Archival + Acoustic + External	ADST-V9TP Pederson disc	27	BE	2018 - 2019	354	180 - 90	H 90 days (240 - 360 s);<br>H 150 days (120 - 240 s);<br>H 104 days (240 - 360 s)
		40	BE	2018 - 2019	339	180 - 90	H 120 days (120 - 240 s);<br>H 239 days (240 - 360 s)
		19	BE	2020 - 2021	400	180 - 90	H 120 days (180 - 200 s);<br>H 280 days (300 - 400 s)
		23	BE	2020 - 2021	425	300 -90	H 70 days (180 - 280 s);<br>H 355 days (300 - 400 s)
